# Influence of Etchants on Etched Surfaces of High-Strength and High-Conductivity Cu Alloy of Different Processing States

**DOI:** 10.3390/ma17091966

**Published:** 2024-04-24

**Authors:** Jinyang Fang, Qingke Zhang, Xinli Zhang, Feng Liu, Chaofeng Li, Lijing Yang, Cheng Xu, Zhenlun Song

**Affiliations:** 1 School of Materials Science and Engineering, Jiangxi University of Science and Technology, Ganzhou 341000, China; fangjinyang@nimte.ac.cn; 2 Key Laboratory of Advanced Marine Materials, Ningbo Institute of Materials Technology and Engineering, Chinese Academy of Sciences, Ningbo 315201, China; yanglj@nimte.ac.cn (L.Y.); xucheng@nimte.ac.cn (C.X.); songzhenlun@nimte.ac.cn (Z.S.); 3 Ningbo Kangqiang Electronics Co., Ltd., Ningbo 315105, China; zhangxl@kangqiang.com (X.Z.); lcf@kangqiang.com (C.L.); 4 Ningbo Xingye Shengtai Group Co., Ltd., Ningbo 315336, China

**Keywords:** Cu alloy, etching, ferric chloride, copper chloride, surface morphology, roughness

## Abstract

With the continuous integration of semiconductor devices, the requirements of the size accuracy and surface quality of etched lead frames are stricter. The etchant is a key factor in the etching process and etched surface quality, while the effects of the difference in etchants on the etched surface morphology of Cu alloy have not been directly studied. In this study, aqua regia, acidic FeCl_3_ and two CuCl_2_ solutions were used as etchants, and different CuCrSn specimens were etched and characterized. The results show that the etching rate in aqua regia is high, and the grain orientation, grain boundary (GB) and dislocations have significant influences on the local etching rate. The preferential etching of some atomic planes forms steps between the grains with different orientations, and preferential etching around the GB and dislocation group forms grooves, resulting in high surface roughness. For the surfaces etched by the FeCl_3_ and CuCl_2_ etchants, the steps and grooves are blurred; thus, they are less rough. The CuCrSn alloy surface etched by the aqua regia is clean, with little Cr-rich particles, while high-density Cr-rich particles remain on the surfaces etched by the FeCl_3_ and CuCl_2_ etchants. For the same kind of etchant, the ion concentration can affect the etching mechanism, rate and the etched surface morphology.

## 1. Introduction

Lead frames are widely used for electrical connections between chips and external circuits in semiconductor packaging. With the increased integration of semiconductor devices, the requirements of the size accuracy and surface quality of lead frames are becoming more and more strict [1]. At present, high-precision lead frames are mainly processed by etching [2,3], and the materials are high-strength and high-conductivity Cu alloys [4,5]. Etching is a precise material reduction method which selectively dissolves the unnecessary part of the alloy strips to obtain devices with a specific size and shape. Therefore, both the etchant and microstructure of the alloy will affect the etching chemical reaction and the etched surface.

Cu alloy strips can be processed by a variety of etchants with different etching characteristics [6]. Among these etchants, acidic FeCl_3_ solution is a common etchant composed mainly of ferric chloride and strong acids, which has fast etching rate and suitable for the small-scale etching process of Cu [7,8,9,10]. Compared with the FeCl_3_ etchant, the acidic CuCl_2_ etchant has slower etching rate, while the Cu-dissolving capacity of the acidic CuCl_2_ etchant is higher than that of the FeCl_3_ etchant; thus, it is suitable for the large-scale etching of Cu [11,12,13,14]. Moreover, an alkaline CuCl_2_ etchant composed of ammonia, NH_4_Cl and CuCl_2_ is also widely used in the etching of Cu foils [12,15], but it can change the chemical properties of the resist mask on the lead frame, making it difficult to remove [16]. In addition, some strong acids, such as HNO_3_, H_2_SO_4_ or aqua regia, can also etch Cu alloy [17,18,19]. Recently, the influence of the microstructure and defects on the etching behaviors, etched surface morphology and roughness of Cu alloy etched with aqua regia have been revealed [20,21]. However, to obtain a precisely etched lead frame with a high size accuracy and proper surface roughness, it is necessary to further understand the etching characteristics of Cu alloy in different etchants. The effects of the microstructure of Cu alloy on the etching behaviors of different etchants have not yet been directly investigated.

For the reasons above, in this study, aqua regia, acidic FeCl_3_ and two acidic CuCl_2_ were selected as the etchants. Aqua regia is a typical strong acid etchant similar to the H_2_SO_4_-H_2_O_2_ and HNO_3_ etchants, which can be used to clean etched surfaces that can be easily observed; thus, it was selected to be compared with the widely used acidic FeCl_3_ and acidic CuCl_2_ etchants. CuCrSn alloy was selected to be the experimental material based on some earlier investigations, which is a new high-strength and high-conductivity CuCr series [22,23]. CuCrSn alloy specimens of different processing states were firstly etched using the above etchants, and then the etched surface morphologies were observed, and the surface roughness were measured. Based on this, the etching behaviors of CuCrSn alloy in the different etchants and the influence of the microstructure of Cu alloy on the etched surface were discussed. It is expected that this work can provide a reference for controlling the etching accuracy and etched surface of lead frames.

## 2. Materials and Methods

### 2.1. Preparation of Materials

In this study, a CuCrSn specimen was smelted in an atmosphere protection furnace with Ar of 99.99% purity as the shielding gas. The raw materials were pure Cu (99.99 wt%), Cu-10Cr intermediate alloy and Sn particles with a purity of 99.99 wt%. The designed composition of the alloy was Cu-0.45Cr-0.25Sn (wt%), and the raw materials were weighed according to such a composition. To decrease the burning loss during the smelting process, the furnace was first heated to 1000 °C, and then the raw materials were put into a graphite crucible. The furnace was continually heated to 1250 °C for 50 min. After that, the liquid alloy was casted in a mold and air cooled to obtain ingots of 10 mm in thickness.

To make the composition more uniform, the CuCrSn cast ingot was homogenized and annealed at 960 °C for 6 h, and then cooled in the furnace. The composition of the ingot was analyzed by inductively coupled plasma emission spectrometry (ICP-OES, Spectro AcroII), and the result shows that the mass fractions of Cr and Sn elements were 0.437% and 0.253%, respectively, which were quite close to the target composition. The homogenized CuCrSn alloy specimen was cold-rolled to 2 mm, and the cold rolling deformation value was 80%. To obtain CuCrSn specimens with different microstructure and grain sizes, the cold-rolled specimens were annealed at 600 °C, 700 °C, 750 °C, 800 °C or 850 °C for 15 min.

### 2.2. Etching and Characterization Methods

The surface microstructure of the cold-rolled and annealed specimens before etching was observed with a metallographic microscope (NMM-800RF, Ningbo Yongxin Optics Co., Ltd., Ningbo, China). For all the CuCrSn specimens, the cross sections parallel with the cold rolling direction of the plates were observed. The processing of all the specimens was performed as follows: they were sequentially ground with sandpaper from 500# to 3000#, followed by vibrationally polishing using 2.5 μm- and 0.5 μm-sized diamond polishing agents. To clearly show the grains of the alloy specimens, the proportion of corrosion solution for metallographic etching was 5 g FeCl_3_·6H_2_O + 10 mL concentrated HCl + 100 mL absolute ethanol.

The composition of acidic FeCl_3_ etchant is 45 g FeCl_3_·6H_2_O + 100 mL deionized water + 10 mL concentrated HCl. Two different CuCl_2_ etchants were used. The composition of the first is 20 g CuCl_2_·2H_2_O + 100 mL deionized water + 15 mL concentrated HCl (CuCl_2_-1), and the composition of the second is 28.5 g CuCl_2_·2H_2_O + 112 mL deionized water + 10 mL concentrated HCl (CuCl_2_-2). The contents of Cu^2+^ and Cl^−^ in the CuCl_2_-1 etchant are 1.0 mol/L and 3.24 mol/L and 1.3 mol/L and 3.38 mol/L in the CuCl_2_-2 etchant, respectively. Aqua regia was made by mixing HNO_3_ and concentrated HCl at 1:3, and it was kept for 1 h after mixing so that the two acids could fully react. All the specimens were etched in the etchants for 3 min. The etched surfaces were ultrasonic cleaned, and then observed with a scanning electron microscope (SEM, Sirion 200, Thermo Fisher, Waltham, USA; EVO18, Carl Zeiss AG, Oberkochen, Germany) and confocal laser scanning microscope (CLSM, Zeiss LMS700), and the surface roughness was also measured using the CLSM. The distribution of elements on the etched surfaces was measured with an energy-dispersive spectrometer (EDS). Table 1 presents the preparation of specimens, the etching and characterization methods.

## 3. Results and Discussion

### 3.1. Grain Structure of Specimens Annealed at Different Temperatures

**Figure 1:**
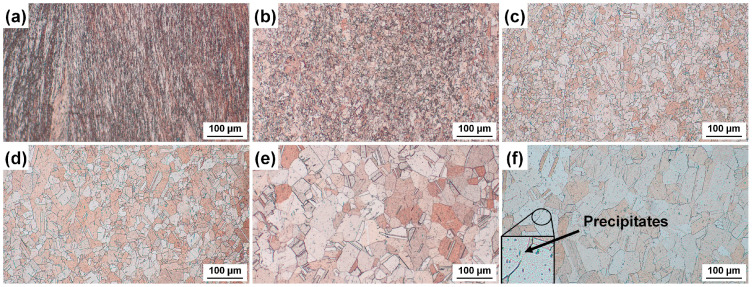
Microstructure of the different CuCrSn specimens: (**a**) the 80% cold-rolled specimen without annealing and the specimens further annealed at (**b**) 600 °C, (**c**) 700 °C, (**d**) 750 °C, (**e**) 800 °C or (**f**) 850 °C for 15 min after cold rolling.

The microstructures of the 80% cold-rolled CuCrSn alloy before and after annealing are presented in Figure 1. Before annealing, the cold-rolled specimens shows a typical rolling texture structure, and high-density deformation defects and grain boundaries (GBs) were observed, as shown in Figure 1a. The cold-rolling process resulted in a large number of dislocations and defects, which decreased the binding energy and became the preferential etching site [24,25]. Meanwhile, grain fragmentation also occurred; thus, the density of the GBs increased significantly. After being kept at 600 °C for 15 min, recrystallization occurred in almost all the area, forming very fine grains, and meanwhile the rolling texture was eliminated (see Figure 1b). As the annealing temperature increased, the size of the grains increased gradually, while the defects in the Cu alloys were basically not observed, as presented in Figure 1c–f, because the annealing treatment can eliminate high-density dislocations and some other defects formed during the cold rolling process. As most of the defects have been eliminated, some precipitation particles can be clearly identified in the specimens annealed at higher temperature. Generally, the deformation texture and defects were eliminated, and recrystallized grains with different size were obtained after annealing at different temperatures.

### 3.2. Surface Morphologies after Etching with Different Etchants

**Figure 2:**
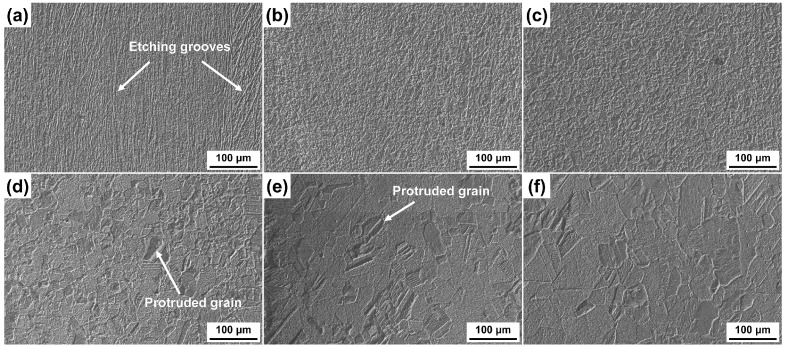
Surface morphologies of the different CuCrSn specimens etched by aqua regia: (**a**) the 80% cold-rolled specimen without annealing and the specimens further annealed at (**b**) 600 °C, (**c**) 700 °C, (**d**) 750 °C, (**e**) 800 °C or (**f**) 850 °C for 15 min after cold rolling.

The surface morphologies of six CuCrSn alloy specimens etched for 3 min in the aqua regia etchant are shown in Figure 2. For the 80% cold-rolled specimens, high-density etching grooves can be observed on the etched surface, as exhibited in Figure 2a, because dislocation groups and new GBs were formed during cold rolling, and the Cu atoms close to these locations were preferentially dissolved. The deeply etched grooves increased the adhesion strength between the molding compound and the etched lead frame. After annealed at 600 °C and 700 °C, the dislocations and the original parallel GBs disappeared, and high-density recrystallized GBs formed, as shown in Figure 2b,c. Although the dissolution of Cu atoms close to the GBs occurred faster, and thus the GBs were etched preferentially, the difference in etching rates between different grains was not very serious, and the etched surface remained flat as the grains were very fine. The etched surface morphologies of specimens annealed at higher temperature are shown in Figure 2d–f, in which the grain size increases. As the dissolution rates of different atomic planes were different, the etching rates of grains with different orientation are very different [21]. As a result, some protruded grains emerged because the etching rates of these grains are lower, and some steps formed at the GBs. Meanwhile, as the density of the GBs decreases, the effect of GBs on the etching became less serious. In addition, it was found that the microscopic etched surface morphologies of different grains are very different, which also basically dominated by the orientation of these grains, and the mechanisms have been revealed [21]; thus, it will not be deeply analyzed again in this paper.

**Figure 3:**
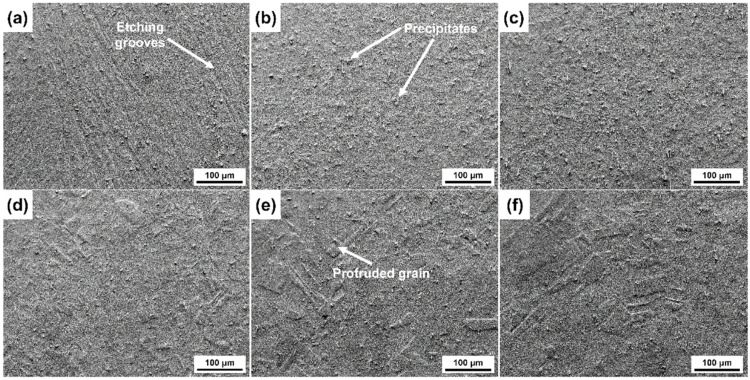
Surface morphologies of the different CuCrSn specimens etched by acidic FeCl_3_ etchant: (**a**) the 80% cold-rolled specimen without annealing and the specimens further annealed at (**b**) 600 °C, (**c**) 700 °C, (**d**) 750 °C, (**e**) 800 °C or (**f**) 850 °C for 15 min after cold rolling.

The surface appearances of the CuCrSn specimens etched for 3 min in the FeCl_3_ etchant are shown in Figure 3. It can be found that effects of the dislocation groups and grain orientation and on etching can still be seen. For the cold-rolled specimen shown in Figure 3a, parallel etching grooves at the dislocation groups can be observed, whereas compared with the specimen surface etched by aqua regia, the etched grooves are blurred. In Figure 3b,c, although recrystallization has occurred, it is difficult to distinguish the GBs; thus, it can be predicted that the influence of GBs on etching became negligible. As the annealing temperature increased, the grain size became larger, and the difference in etching rate due to the different grain orientations became more and more obvious. In Figure 3d, a clear height difference between the neighboring grains can already be seen, and this difference is more obvious in Figure 3e,f. However, the steps at the GBs are not so sharp as those on the surface etched by aqua regia. In addition, the microstructures of the etched surfaces of different grains with different orientations are very similar, and some residual particles can be found on the etched surfaces.

**Figure 4:**
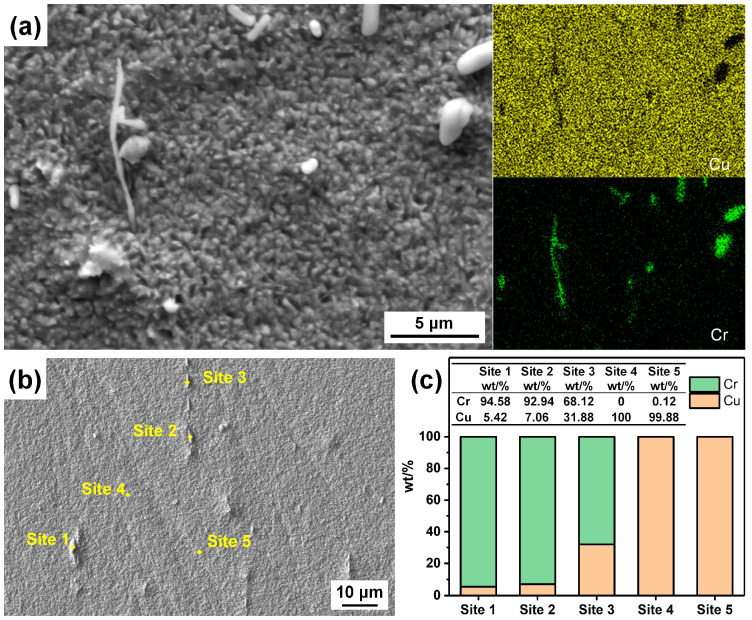
(**a**) Microscopic surface morphology and elemental distribution of the 80% cold-rolled specimen after being etched by the FeCl_3_ etchant, (**b**) sites for the EDS characterization and (**c**) compositions of these sites.

Figure 4 shows the morphology and composition of the particles on the surface etched by FeCl_3_ etchant. In Figure 4, it can be found that the size of the bright particles ranges from one to a few micrometers, and they have different shapes, which can also be observed in Figure 1. Figure 4b,c shows the EDS point analysis sites and the corresponding compositions, which also demonstrate that the particles are Cr-rich, and the rest of the surface is almost pure Cu. The presence of these residual particles could be due to the very low dissolution rate of the Cr-rich phase in the FeCl_3_ etchant. These particles on the surface cannot be removed by ultrasonic cleaning; thus, if they are too large in size, they may be detrimental to the bonding of the molding compound in the subsequent packaging process.

**Figure 5:**
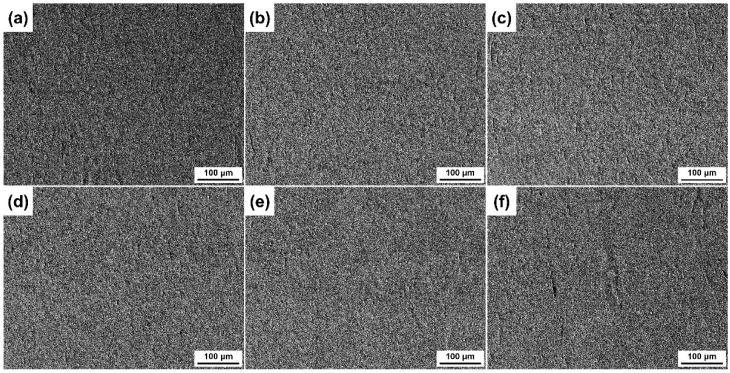
Surface morphologies of the different CuCrSn specimens etched by the CuCl_2_-1 acidic etchant: (**a**) the 80% cold-rolled specimen without annealing and the specimens further annealed at (**b**) 600 °C, (**c**) 700 °C, (**d**) 750 °C, (**e**) 800 °C or (**f**) 850 °C for 15 min after cold rolling.

Figure 5 exhibits the surface morphologies of the CuCrSn specimens etched by the CuCl_2_-1 etchant. As shown in Figure 5a, the dislocation groups and a large number of GBs generated by cold rolling are indistinguishable. With the recrystallization and growth of the grains, the influence of grain orientation on the etched surface morphology is still not obvious, and there is almost no protruded grain on the etched surfaces, as shown in Figure 5b–f. As a result, all the specimens in Figure 5 have very similar smooth morphologies after etching. There are also surface residual particles on the etched surface, but their size is smaller than those in Figure 3.

**Figure 6:**
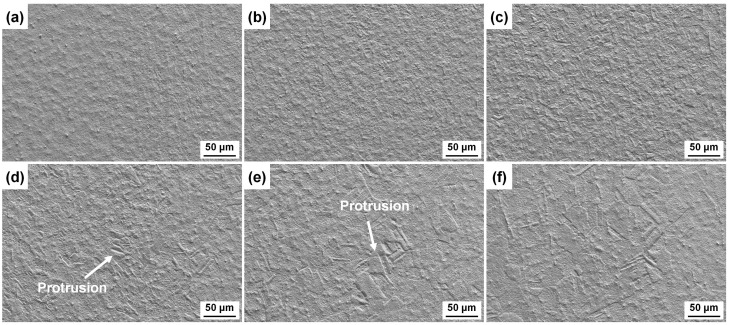
Surface morphologies of the different CuCrSn specimens etched by the CuCl_2_-2 acidic etchant: (**a**) the 80% cold-rolled specimen without annealing and the specimens further annealed at (**b**) 600 °C, (**c**) 700 °C, (**d**) 750 °C, (**e**) 800 °C or (**f**) 850 °C for 15 min after cold rolling.

The surface morphologies of the CuCrSn specimens etched by the CuCl_2_-2 etchant are presented in Figure 6. For the cold-rolled specimens, the dislocation groups and GBs had a little effect on etching, and the surface was flat, as shown in Figure 6a. However, after recrystallization occurred, the effects of grain orientation on the etching rate became visible, and some slight protrusions can be observed (see Figure 6b,c). When the grain size became larger, the protrusions of the etch-resistant grains became quite obvious, as shown in Figure 6d–f, which are different from the surfaces etched with the CuCl_2_-1 etchant, i.e., the grain orientation is more likely to affect the etched surface in the CuCl_2_-2 etchant. For same kind of etchant, the concentrations of ions also affected the etching behavior.

**Figure 7:**
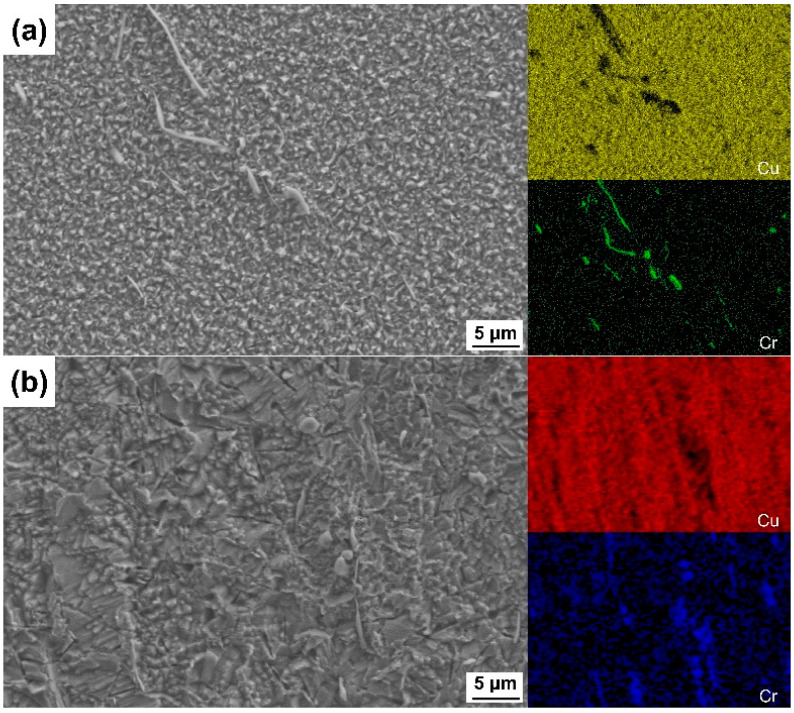
Surface morphologies and elemental distributions of the 80% cold-rolled specimen etched by the (**a**) CuCl_2_-1 and (**b**) CuCl_2_-2 etchants.

Figure 7 shows the surface morphologies and elemental distributions on the surfaces etched by the two CuCl_2_ etchants. For the surface etched by the CuCl_2_-1 etchant, the surface microstructure is very fine, as shown in Figure 7a. Some residual fine Cr-rich precipitates can be seen on the etched surface. Figure 7b shows the surface etched by the CuCl_2_-2 etchant, it seems that it is not so uniform as that of Figure 7a, and the surface is rougher. From the microscopic etched surface morphologies, it can be predicted that the ion concentration of the CuCl_2_ etchant affected the etching process and morphology of the etched surface. With a higher concentration of ions, the etching rate will be higher, and the surface will also become coarser.

### 3.3. 3D Surface Undulations after Etching with Different Etchants

**Figure 8:**
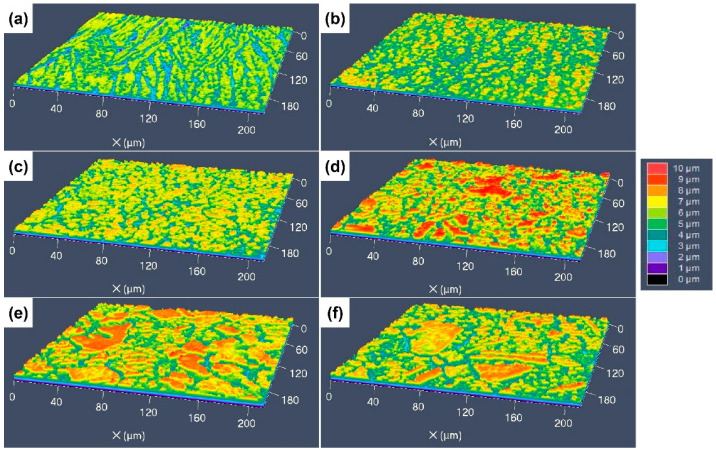
Three-dimensional morphologies of the different CuCrSn specimens etched by aqua regia: (**a**) the 80% cold-rolled specimen without annealing and the specimens further annealed at (**b**) 600 °C, (**c**) 700 °C, (**d**) 750 °C, (**e**) 800 °C or (**f**) 850 °C for 15 min after cold rolling.

The 3D surface morphologies of the specimens etched by the four different etchants were characterized using CLSM to reveal the difference in height between the different areas of the etched surfaces, with different colors indicating the difference in height. Figure 8 shows the 3D morphologies of the specimens etched by aqua regia. For the cold-rolled specimens, the deep grooves around the GBs and dislocation groups can be clearly seen, and the difference in height between the grooves and the surrounding areas is about 3–4 μm. After an annealing at 600 °C, the deepest sites on the etched surface are located at the GBs, as presented in Figure 8b, and the difference in etch rates of different grains is not very obvious. As the annealing temperature increased, some protrusions with a red color appeared, as shown in Figure 8c–f. The obvious difference in the etching rate occurred not only at the GBs and inside the grains, but also between the different grains. Therefore, some red areas can be observed on the etched surfaces, and the height difference in the etched surfaces is about 5–6 μm.

**Figure 9:**
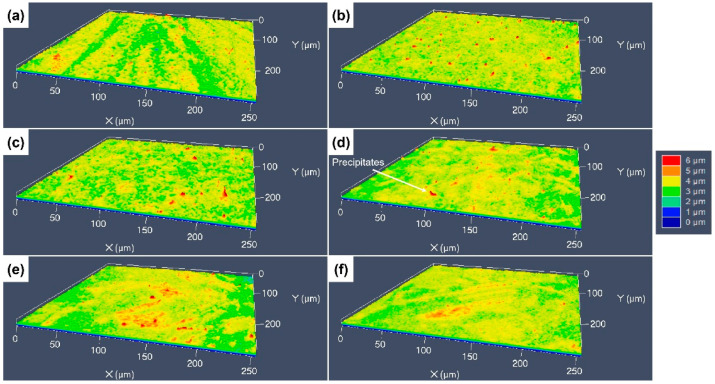
Three-dimensional morphologies of the different CuCrSn specimens etched by the FeCl_3_ etchant: (**a**) the 80% cold-rolled specimen without annealing and the specimens further annealed at (**b**) 600 °C, (**c**) 700 °C, (**d**) 750 °C, (**e**) 800 °C or (**f**) 850 °C for 15 min after cold rolling.

Figure 9 shows the 3D surface morphologies of the specimens etched using the FeCl_3_ etchant. For the original cold-rolled specimen, it seems that the etch grooves at the dislocation groups become blurred, but still exist. The depth of the etching grooves is only 1 μm, which is much shallower than that on the surface etched by aqua regia, and the width reaches to about 20 μm. In Figure 9b,c, some red protrudent particles can be seen, which are the residual Cr-rich precipitations. The height of these particles raised more than 2 μm. With increasing grain size, the protrudent etch-resistant grains can be observed, as presented in Figure 9d–f, but the height of the protrusions is only 2–3 μm.

**Figure 10:**
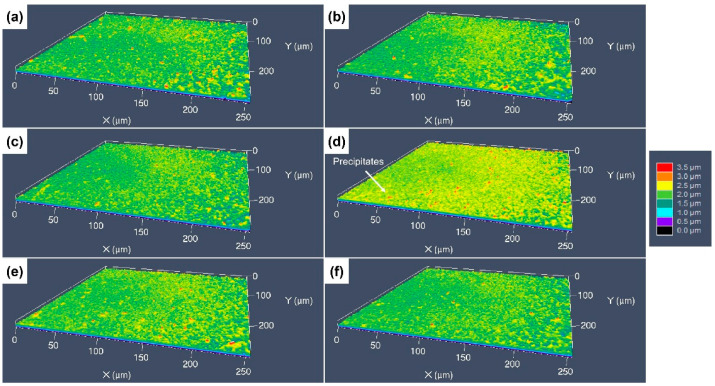
Three-dimensional morphologies of the different CuCrSn specimens etched by the CuCl_2_-1 etchant: (**a**) the 80% cold-rolled specimen without annealing and the specimens further annealed at (**b**) 600 °C, (**c**) 700 °C, (**d**) 750 °C, (**e**) 800 °C or (**f**) 850 °C for 15 min after cold rolling.

**Figure 11:**
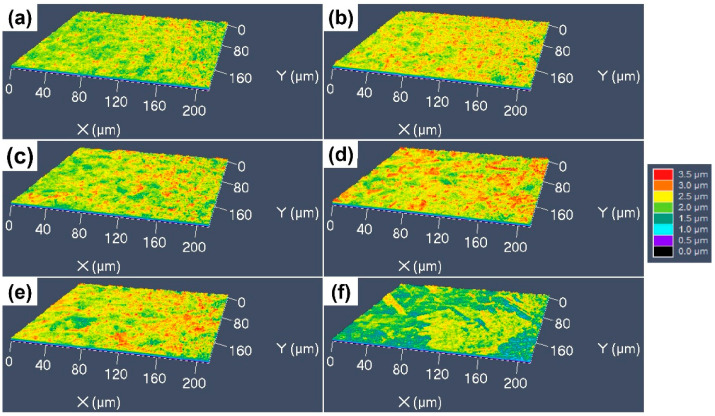
Three-dimensional morphologies of the different CuCrSn specimens etched by the CuCl_2_-2 etchant: (**a**) the 80% cold-rolled specimen without annealing and the specimens further annealed at (**b**) 600 °C, (**c**) 700 °C, (**d**) 750 °C, (**e**) 800 °C or (**f**) 850 °C for 15 min after cold rolling.

The 3D surface morphologies of the specimens etched by the CuCl_2_-1 etchant are shown Figure 10. It can be found that all the surfaces are very flat, with no obvious protrusions, as shown in Figure 10a–c; the dislocation groups, GBs and grain orientation have a little influence on the etching surface. For the specimens with coarser grains, some fine red particles can still be found on the etched surfaces (see Figure 10d–f), which is consistent with the SEM images. Figure 11 shows the 3D surface morphology of the specimens etched by the CuCl_2_-2 etchant, for which the surface fluctuation is larger than that etched by the CuCl_2_-1 etchant. For the cold-rolled specimens, the deformation defects had a little influence on the etched surface, but the grain orientation had an obvious influence and formed some protrudent grains.

### 3.4. Surface Roughness

**Figure 12:**
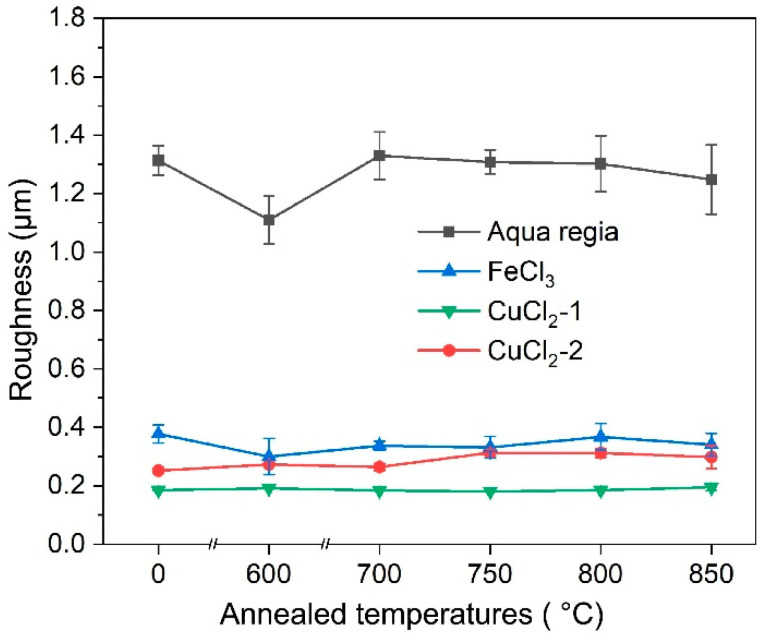
Surface roughness of the CuCrSn alloy specimens etched by the 4 different etchants.

**Figure 13:**
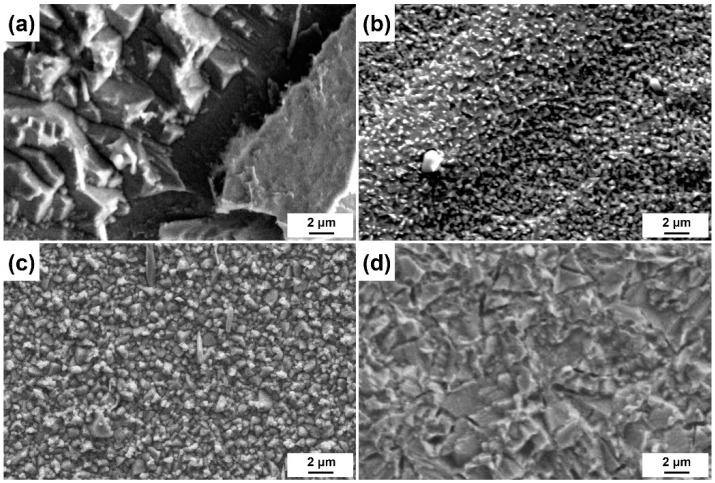
Microscopic surface morphologies of the specimens etched by the (**a**) aqua regia, (**b**) FeCl_3_, (**c**) CuCl_2_-1 and (**d**) CuCl_2_-2 etchants.

The obtained surface roughness of the specimens etched by different etchants is shown in Figure 12. For the surfaces etched with aqua regia, they are significantly rougher because preferential etching at the GBs and dislocation groups in aqua regia is very serious. However, it seems that the FeCl_3_ and CuCl_2_ etchants are insensitive to these defects; thus, the GBs and defects are blurred and almost indistinguishable on the etched surface. It was revealed that the grain size affects the roughness of the CuCrSn alloy surface etched by aqua regia [21]. For FeCl_3_ and the two CuCl_2_ etchants, recrystallization and grain growth has a little influence on the etched surface roughness, although the etched surface morphology is affected by the grain structure, and the annealing temperature–surface roughness curves show almost no fluctuations. Figure 13 shows the microscopic surface morphologies of the CuCrSn specimens etched by the four etchants, with the same magnification factor, in which the difference in surface roughness is very clear.

### 3.5. Discussion

From the above experimental results, it can be found that the grain structure, GBs and dislocation groups of the CuCrSn alloy can affect the etched surface morphology, whereas the influence levels of these factors are quite different in the different etchants. In aqua regia, the main substance that reacts with Cu is concentrated nitric acid, and Cl^−^ easily forms complexes with Cu^2+^, which decreases the reduction potential of Cu and accelerates the reaction. The reaction equation is as follows [26]:

(1) $$Cu\left(s\right)+4H{NO}_{3}\left(aq\right)=2H_{2}O(l)+2NO_{2}(g)+Cu{\left({NO}_{3}\right)}_{2}(aq)$$

During the etching process, Cu is directly oxidized into Cu^2+^, and then rapidly dissolved. As the reactivity of the atoms near defects and the GBs is higher, which accelerates the dissolution rate, the aqua regia etchant is strongly influenced by the defects, GBs and crystal orientation. Moreover, the bonding energies between the different atomic planes are different, resulting in a difference in the etching rates of grains of different orientations and very fine step morphologies within the etched surface of the grains [21].

For the FeCl_3_ etchant, the Fe^3+^ ions on the surface of the alloy oxidize Cu atoms into Cu^+^, and the resulting CuCl is insoluble in the solution and will cover the surface of the alloy. Then, the CuCl can be further oxidized by Fe^3+^ into CuCl_2_; the reaction equation is as follows [16,27,28]:

(2) $$Cu(s)+{FeCl}_{3}(aq)=CuCl(s)+{FeCl}_{2}(aq)$$

(3) $$CuCl(s)+{FeCl}_{3}(aq)={CuCl}_{2}(aq)+{FeCl}_{2}(aq)$$

For etching in the FeCl_3_ etchant, the Cu atoms at the surface are in direct contact with Fe^3+^ in the beginning; thus, the etching rate is the highest. Since the dissolution rate of the CuCl is lower than the generation rate, CuCl film is formed on the alloy surface, which prevents Fe^3+^ from making contact with the alloy surface and hindering Reaction (2). As a result, the generation rate of the CuCl film decreases until the generation and dissolution of CuCl, achieving dynamic equilibrium, and the etching rate begins to stabilize [29,30]. Since there is a CuCl film that affects the etching mechanism, the surface morphology etched with the FeCl_3_ etchant is obviously different from that etched with aqua regia. The effects of the defects, GBs and grain orientation on etching can still be observed, but are suppressed to a large extent.

The reaction processes of the CuCl_2_ etchant with the Cu alloys are as follows [11,12,13]:

(4) $$Cu{Cl}_{2}(aq)+Cu(s)={Cu}_{2}{Cl}_{2}(s)$$

(5) $${Cu}_{2}{Cl}_{2}(s)+4{Cl}^{-}=2{\left[Cu{Cl}_{3}\right]}^{2-}$$

The Cu^2+^ ions first oxidize the Cu atoms into Cu^+^, which also forms CuCl films. Unlike the FeCl_3_ etchant, the CuCl film can only be dissolved through the adsorption of Cl^−^ to form a complex, and the dissolution rate of the CuCl film in the CuCl_2_ etchant is lower than that in the FeCl_3_ etchant. 

**Figure 14:**
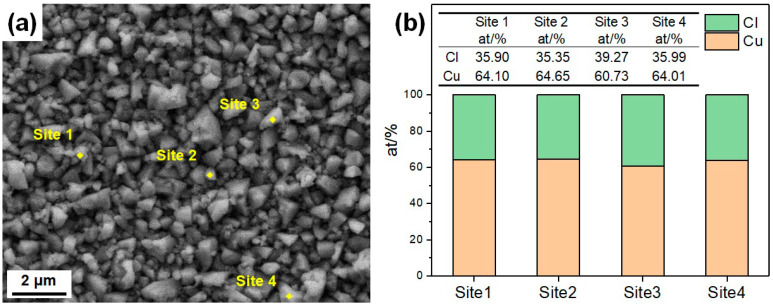
(**a**) Characterization sites and (**b**) the corresponding compositions of a specimen etched by the CuCl_2_-1 etchant, with no ultrasonic cleaning after etching.

Figure 14 shows the characterization sites and the corresponding compositions of a specimen etched by the CuCl_2_-1 etchant, with no ultrasonic cleaning after etching, which demonstrates that the etched surface before ultrasonic cleaning is almost covered by CuCl. Therefore, the influence of the CuCl film on the etching process is more serious, while the influence of the defects, GBs and grain orientation on the etching process is much weaker, so the consistency of the etched surface in the CuCl_2_ etchant is better, and the surface is less rough. The CuCl_2_-2 etchant has a higher content of Cl^−^ and Cu^2+^, making it more erosive to the CuCl film and the etching is less uniform. As the difference in etching rates of CuCl_2_ in the matrix and the precipitates is low, the residual precipitation particles on the surfaces etched by the CuCl_2_ etchant are smaller in size.

## 4. Conclusions

The etching surface of a set of the CuCrSn alloy specimens with different microstructures and grain sizes by four different etchants (aqua regia, FeCl_3_, CuCl_2_-1 and CuCl_2_-2) were characterized in this study. The main conclusions are as follows:

1. The etching rate in aqua regia is high, and the grain orientation, GBs and dislocations have significant influences on the etching rate, the height difference between the etch-resistant grains and the surrounding grains of about 5–6 μm. The preferential etching of some atomic planes forms steps at some GBs, and preferential etching around the GB and dislocation group forms grooves. For etching with the FeCl_3_ and CuCl_2_-2 etchants, such steps and grooves become blurred and almost invisible on the surface etched by the CuCl_2_-1 etchant.
2. The CuCrSn alloy surface etched by aqua regia is clean, with very little Cr-rich particles. For the specimens etched with the FeCl_3_ and CuCl_2_ etchants, high-density Cr-rich particles remained on the surfaces; thus, etching in these two etchants is more likely to be affected by the alloy composition and the precipitations.
3. Due to the serious difference in etching rate at the different locations, the surface roughness of the specimens etched by aqua regia is about 1.27 μm. There is a little fluctuation on the surfaces etched with the FeCl_3_ and CuCl_2_ etchants; thus, the surface roughness are only around 0.2~0.3 μm. For the same kind of etchant, the ion concentration can affect the etched surface morphology, although not fundamentally.

## Figures and Tables

**Table 1:** The preparation of specimens, etching and characterization methods in this study.

Alloy Composition	Treatments of Specimens	The Four Etchants	Characterization
Cu-0.437Cr-0.253 (wt%)	80% cold-rolled; annealed at 600 °C, 700 °C, 750 °C, 800 °C and 850 °C for 15 min after 80% cold-rolling	Aqua regia	metallographic microscope SEM CLSM EDS
		Acidic FeCl_3_ etchant: 45 g FeCl_3_·6H_2_O + 100 mL H_2_O + 10 mL concentrated HCl	
		CuCl_2_-1: 20 g CuCl_2_·2H_2_O + 100 mL H_2_O + 15 mL concentrated HCl	
		CuCl_2_-2: 28.5 g CuCl_2_·2H_2_O + 112 mL H_2_O + 10 mL concentrated HCl	

## Data Availability

Data are contained within the article.
